# Role of Personalized Nutrition in Chronic-Degenerative Diseases

**DOI:** 10.3390/nu11081707

**Published:** 2019-07-24

**Authors:** Laura Di Renzo, Paola Gualtieri, Lorenzo Romano, Giulia Marrone, Annalisa Noce, Alberto Pujia, Marco Alfonso Perrone, Vincenzo Aiello, Carmela Colica, Antonino De Lorenzo

**Affiliations:** 1 Section of Clinical Nutrition and Nutrigenomic, Department of Biomedicine and Prevention, University of Rome Tor Vergata, Via Montpellier 1, 00133 Rome, Italy; 2 School of Specialization in Food Sciences, University of Rome Tor Vergata, Via Montpellier 1, 00133 Rome, Italy; 3 PhD School of Applied Medical-Surgical Sciences, University of Rome Tor Vergata, Via Montpellier 1, 00133 Rome, Italy; 4 UOC of Internal Medicine-Center of Hypertension and Nephrology Unit, Department of Systems Medicine, University of Rome Tor Vergata, via Montpellier 1, 00133 Rome, Italy; 5 Department of Biomedicine and Prevention, University of Rome Tor Vergata, Via Montpellier 1, 00133 Rome, Italy; 6 Division of Cardiology, University of Rome Tor Vergata, Via Montpellier 1, 00133 Rome, Italy; 7 Department of Medical and Surgical Sciences, University of Magna Græcia, Viale Europa, Germaneto, 88100 Catanzaro, Italy; 8 Institute of Molecular Bioimaging and Physiology, National Research Council, Organizational Support Unit of Germaneto, University “Magna Graecia” of Catanzaro, Campus “Salvatore Venuta”, 88100 Catanzaro, Italy

**Keywords:** nutrigenetic, nutrigenomic, personalized nutrition, epigenetic, Mediterranean diet, obesity, gut microbiota

## Abstract

Human nutrition is a branch of medicine based on foods biochemical interactions with the human body. The phenotypic transition from health to disease status can be attributed to changes in genes and/or protein expression. For this reason, a new discipline has been developed called “-omic science”. In this review, we analyzed the role of “-omics sciences” (nutrigenetics, nutrigenomics, proteomics and metabolomics) in the health status and as possible therapeutic tool in chronic degenerative diseases. In particular, we focused on the role of nutrigenetics and the relationship between eating habits, changes in the DNA sequence and the onset of nutrition-related diseases. Moreover, we examined nutrigenomics and the effect of nutrients on gene expression. We perused the role of proteomics and metabolomics in personalized nutrition. In this scenario, we analyzed also how dysbiosis of gut microbiota can influence the onset and progression of chronic degenerative diseases. Moreover, nutrients influencing and regulating gene activity, both directly and indirectly, paves the way for personalized nutrition that plays a key role in the prevention and treatment of chronic degenerative diseases.

## 1. Introduction

Human nutrition is a relatively new, biochemically-based science. Although its roots are entrenched in centuries of learning in medicine, the scientific study of food and human requirements began less than 100 years ago. The field of human nutrition is progressing constantly. From geographic epidemiologic studies, it is proceeding to inform a better understanding of individuals and their bioanalytical profiles and it has achieved integration with pharmacology [1].

Asian and pre-Christian Greek cultures developed the idea that since some foods made illness, other might make health. Early medicine of both cultures included many foods as remedies for physical performance or disease care.

Already in the fourth century B.C., Hippocrates had highlighted how food could be our first medicine and how a balanced diet could help maintain health. Today we know that the way we eat depends on our specific genetic characteristics and that it can modulate gene expression, protein synthesis and metabolic and cellular processes [2]. During the Naturalistic Era (400 B.C.–1750 A.C.), it was established with greater force that the human diet could play a fundamental role in preventing and treating certain diseases. In the course of the Chemical-Analytical Era, we have learnt more about the specific composition of foods, while during the Biological Era we have gained better knowledge on the metabolic pathways and the role of micronutrients and macronutrients on human health.

The rapid progress of molecular biology determined a revolution in human nutrition as in other medical sciences so impressive that can be defined “Copernican”. This revolution lead to the birth of a new discipline, i.e., molecular nutrition. Necessity of exploring nutrients’ role and mechanisms of action, interpreting the molecular and cellular basis of individual variations, understanding genotype-environmental interactions, focalizing on relationships between chronic-degenerative diseases and nutritional anamnesis, together brought human nutrition and medicine to research food production optimization for specific populations given genetical, ethnical, cultural and economical settings [3].

In the second part of the twentieth century, there was an enormous growth in knowledge and understanding of the mechanisms underlying certain diseases and their relationship with dietary components. This has laid the foundation for food education and nutritional counselling [4].

Traditional nutritional research has contributed significantly to modern biomedicine and has promoted prolongation of life expectancy [5].

Furthermore, the success of the Human Genome Project and refined molecular biology instruments allowed development of a dichotomy [6].

On one side, nutritional genomics, i.e., the interaction between genetic sequences and nutrients; from the other side, metabolomics, defined as the study of metabolites or “metabolomes”.

Molecular nutrition, the birth of a DNA-nutrient database and the acquisition of specific biomarkers, will probably lead to the formulation of a diet in which the nutrient is not only food, but, through biotechnological nourishment, it’ll be considered the first actor in disease prevention, becoming a drug itself [6].

Young et al. [7] defined a nutrient as “a fully characterized (physical, chemical, physiological) constituent of a diet that serves as a significant energy yielding substrate, or a precursor for the synthesis of macromolecules or of other components needed for normal cell differentiation, growth, renewal, repair, defense and/or maintenance, or a required signaling molecule, cofactor or determinant of normal molecular structure/function and/or promoter of cell and organ integrity”.

Interrelations between bioactive food components (BFC) and cellular events were notable. Changes are evident to various molecular levels, involving DNA (i.e., nutrigenetics), in his turn object of pre-transcriptional modifications (i.e., metilations and epigenetics) [8,9] involving the transcript, mRNA (i.e., nutrigenomics) [10] and proteins (i.e., proteomics) [11].

Since nutrition plays a fundamental role in the prevention of certain diseases, scientific research has focused its attention on the possibility of developing a nutrition that takes into account the single individual and his/her genotype, in order to improve the impact of diet on health status. The concept of molecular nutritional research has been defined as “the science that studies the effects of nutrients, food and its components, on the whole physiology and on the state of good health at the molecular and cellular level”. The detailed determination of the molecular mechanisms underlying the state of health and disease represents a great potential for the promotion of health itself, possibly reducing the incidence of mortality and morbidity [12].

The increasing participation of patients in their own health, combined with a more systemic view of healthcare, is leading to an ever more personalized approach to medicine, whose role will be to integrate scientific discoveries with clinical patient care [13].

Overall, these concepts have paved the way to a new medical paradigm known as P4 medical care (based on a predictive, preventive, personalized and participatory approach), which uses the tools of systems medicine to quantify health status and demystify pathology. This approach manages and integrates heterogeneous and patient-specific molecular, clinical and anamnestic data in order to achieve individual well-being [13].

**Figure 1:**
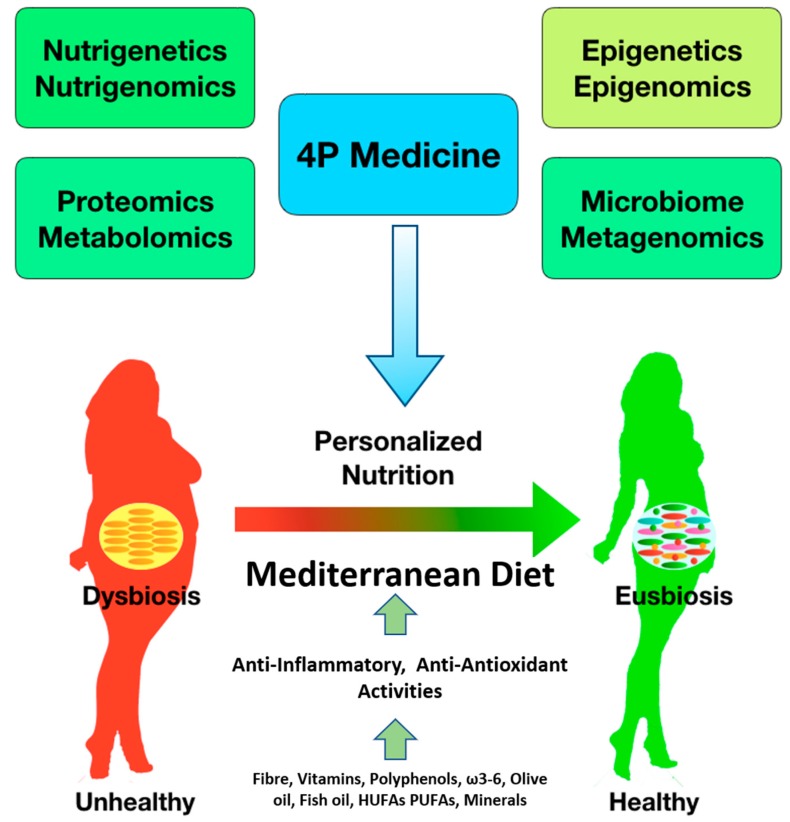
4P medicine, in the era of -omics sciences; to switch from an unhealthy condition of dysbiosis to a healthy condition of eusbiosis. 4P: Predictive, personalized, preventive and participatory; HUFAs: h-3 highly unsaturated fatty acids; PUFAs: polyunsaturated fatty acids.

There are many scientific results demonstrating that diet is a key modifiable factor able to influence the incidence of many chronic diseases. Food, in fact, contains several biologically active substances with health benefits. New -omics technologies have recently shed light on the molecular and cellular effects of nutrients in the individual (Figure 1). This has permitted the customization of nutritional interventions, as well as the production of custom-made foods, based on the physiological, genetic, ethnic, cultural and economic background of the individual [7,14].

The knowledge acquired from the sequencing of human genomes has led to the consolidation of a new molecular dimension of medicine, in particular, known as the field of “predictive medicine”.

By using the unique genetic information that can be obtained in each individual, this approach is capable of anticipating the estimated risk of developing certain diseases in a subject’s lifetime [15].

The aim of this review is to give indications on the -omic sciences application in the personalization of the Mediterranean Diet (MD) and in the treatment in chronic-degenerative disease (CDDs).

## 2. Methods

### Search Strategy and Study Selection

**Figure 2:**
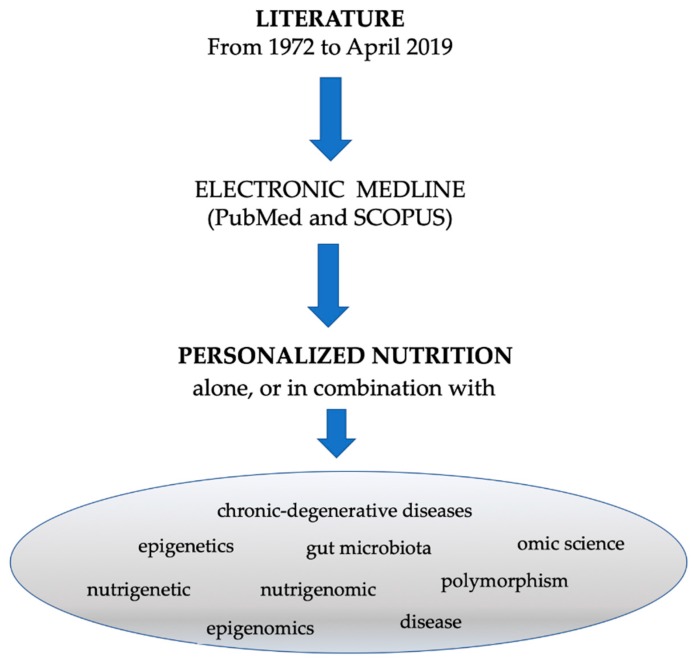
Flow diagram of search strategy.

Literature database of articles from 1972 to April 2019, by searching the electronic MEDLINE via PubMed and SCOPUS was performed. Relevant keywords to term personalized nutrition were analyzed alone or in association with other terms as “chronic-degenerative diseases”, “omic science”, “nutrigenetic”, “nutrigenomic”, “epigenetics”, “epigenomics”, “health”, “disease”, “gut microbiota” and “polymorphism” (Figure 2).

The reference list of referred articles were manually performed and the literature search was limited to the English language.

## 3. From Health to Disease Status

The progress from a healthy to a diseased phenotype is a slow mechanism that can be attributed to changes in gene and/or protein expression. The main chronic diseases that have spread in the last century due to dietary variations, which also have an effect on gene expression, are as follows: obesity, metabolic syndrome, osteoporosis, type 2 diabetes mellitus (T2DM), cardiovascular diseases (CVD), chronic kidney disease, neurodegenerative diseases and cancer. All of them are multifactorial diseases, because of their complex interactions between different genes/proteins and environmental factors, like nutrients and bioactive food components.

The metabolic phenotype can be defined by the following relation [14]:

*Genotype* + *Environment* + (*Genotype* × *Environment*)

which underlines the gene-nutrient interaction that includes the environment, capable of determining the susceptibility of individuals to develop illnesses.

By gene-environment interaction, we imply the different effects that a single environmental exposure can have on the risk of developing a diseas, in people with different genotypes.

Therefore, as a long-term factor, the diet reveals aspects of influence on general well-being. To date, some aspects have been discovered, while others still remain unknown [16,17].

The limit of the study of the environmental and genetic factors indicated above, is that they are ever-changing and potentially indefinite in number, which can give rise to statistical problems and the generation of spurious results. Furthermore, it is difficult to deal with statistical confounding factors, because it is not possible to eliminate them if more genes and environmental factors are involved. For this reason, a new admixture mapping technique has been developed, which can be used to both calculate the percentage of descent of an individual, as well as highlight the genes involved in the risk of diseases in different populations [18].

The incidence of obesity and related diseases is rapidly increasing, more than 100-fold over the last three decades without showing the slowdown [19]. Obesity represents a symptom of about 40 monogenic diseases or chromosomal abnormalities [20]. Melanocortin- 4 receptor (MC4R) deficiency is the most common monogenic form of obesity, affecting from 0.5% to 4% of obese children [21,22]. The lack of MC4R, that is involved in the leptin pathway, is responsible for intense hunger in children, but the effect decreases in adolescence [23]. However, for the majority of severely obese individuals [24] the genetic susceptibility to obesity may depend on the cumulative effects of numerous genetic variants in a ‘‘polygenic’’ model (Table 1) [25]. More than 600 genes and DNA regions have been associated with human obesity, as defined in the genome-wide association study (GWAS) [26], even if with conflicting results [27]. In recent decades, the involvement of many genes and polymorphisms in the pathogenesis of overweight/obesity have been hypothesized, such as: tumor necrosis factor-α (TNFα), interleukin (IL)-6, methylenetetrahydrofolate reductase (MTHFR) and fat mass and obesity-associated (FTO) gene. The main association was noted for a common variant at the FTO locus [28]. FTO is fundamental in energy homeostasis, as it is highly expressed in brain regions involved in the control of nutrition and energy expenditure. Furthermore, the expression of FTO in the hypothalamus is modulated by fasting and limited access to food [29], moreover its exposure to a high-fat diet regulates the expression of FTO, at hypothalamic level. In vitro studies have shown that the deprivation of glucose and amino acids decreases the expression of FTO [30]. High levels of adiposity and increased body mass index (BMI) are associated with polymorphisms present on the first intron of the FTO gene. Homozygosity for the A allele of FTO rs9939609 phenotypically correlates with an increase in BMI and adiposity and also increases the risk of obesity by 1.7 times when compared to subjects homozygous for the T allele [31]. Subjects with an AA genotype have alterations of appetite and increased food consumption, probably in function of an altered synthesis and secretion of ghrelin [32]. Furthermore, the C677T polymorphism in the MTHFR gene and the A66G polymorphism in the MTRR (methionine synthase reductase) gene were evaluated as potential candidates responsible for an increased risk of obesity [33]. Both C677T MTHFR and A66G MTRR polymorphisms lead to high homocysteine levels and low folate concentrations, which are observed in overweight/obese subjects [34]. In most of the populations studied, the C677T MTHFR mutation cannot be considered as a single genetic risk factor for CVD, but nevertheless it remains of great interest for its influence on homocysteine levels [35].

TNFα plays a key role in the inflammatory processes that are present in obesity, dyslipidaemia, insulin resistance and cardiometabolic disease [36]. TNFα mRNA levels present in human skeletal muscle and adipose tissue correlate positively with BMI, fat mass and insulin levels and negatively with lipoprotein lipase activity [37,38]. A study conducted on obese rats with insulin resistance, showed that the neutralization of TNFα with a soluble fusion protein, IgG-tumor necrosis factor receptor (TNFR), increases insulin sensitivity and restores plasma insulin values close to normal levels [39,40]. The G/A 308 TNFα polymorphism is independently correlated with fasting glucose concentration, post-prandial triacylglycerol levels and BMI in response to a fat-rich meal [41]. Fernández-Real et al. [42] suggested the existence of a board TNF-leptin: an anomaly in the tumor necrosis factor receptor (TNFR)-2 gene could explain the link between obesity, insulin resistance and increased leptin levels. Subjects with TNFα-disrupted leptin have higher BMI and elevated leptin levels, with disproportionately higher levels of soluble TNFR2.

IL-6 is a cytokine that regulates immunity and induces the acute phase of inflammation. It is released from adipose tissue and its serum concentrations are related to adiposity indices.

A common G174C polymorphism (rs603573) in the promoter of the human IL-6 gene regulates the transcription of this gene. The G allele is more common in lean subjects and subjects with the G174C or G174G genotype have a significantly higher energy expenditure than those with the C174C genotype [43]. IL-6 can influence energy expenditure, improving the stimulation of sympathetic neurons [44].

Weight loss in pathologically obese individuals subjected after laparoscopic adjustable gastric banding (LAGB) may not reach the expected results. It is thought that this may be due to a failure of adherence to the dietary treatment, but in reality, this may depend on the genetic background of each individual. Patients with the G174G or G174C genotype undergoing LAGB, tend to lose weight more easily than others despite following the same dietary treatment [45,46]. Moreover, from the genome-wide polygenic score (GPS) distribution for obesity, we cannot omit the contribution given by the pathways related to appetite regulation, fat storage and alteration of intestinal microbiota [47,48].

The GPS, a new polygenic predictor capable of discriminating among 2.1 million common genetic variants, was recently used to determine the susceptibility to obesity [49]. Furthermore, it is possible to find a strong discrepancy between the GPS and the phenotype, which depends mostly from the environment compared to the genetic mutation, as it happens in subjects who manage to maintain normal weight despite an unfavorable GPS or who develop high obesity despite a favorable GPS [48,50].

To investigate the interactions between genes and nutrients and improve the health of individuals with personalized diets, “-omic” science has been introduced. This science helps to establish guidelines for each subgroup based on phenotype and genotype [51,52,53].

## 4. The -Omic Science

The -omic science is a recent discipline capable of identifying pools of molecules (such as ions, nucleic acids, protein enzymes etc.) in specific biological samples like urine, saliva, cerebrospinal fluid (CSF), serum etc. [54]. Molecular changes may occur at various levels, involving DNA (i.e., nutrigenetics) [33], mRNA and transcription (i.e., nutrigenomics) [10], proteins (i.e., proteomics) [11], low molecular weight metabolites (i.e., metabolome and so metabolomics) [55] and inducing pre-transcriptional modifications (i.e., methylations and epigenetics) [3,8,9].

## 5. Nutrigenetics and Nutrigenomics

The aim of the Human Genome Project was to determine the sequence of nitrogenous base pairs that form human DNA and to identify and map the genes of the human genome [15]. Single nucleotide polymorphisms (SNPs) are genetic changes that occur in 1% of the population. An individual’s risk of developing a disease can be influenced by these SNPs. SNPs can also alter the synthesis and function of proteins, leading to alterations in nutrient metabolism and requirements.

Nutrigenetics can be defined as the part of nutritional genomics that attends to the study of genetic variations among subjects, with regards to their clinical response to specific nutrients (Table 2) [52].

Nutrigenetics studies the relationship between eating habits, changes in DNA sequence and the development of nutrition-related diseases. Numerous studies have highlighted how nutrients can affect the human genotype, determining its state of health or disease [56,57]. These discoveries have permitted the development of personalized dietary-nutritional plans.

Genetic variants in an individual’s genome can increase their likelihood of developing several CDDs such as obesity, T2DM and cancer. In T2DM, genetic factors can increase the risk of onset the disease, while not directly inducing it. Studies have shown how the genetic predisposition to T2DM can be partially or completely abolished by positive lifestyle changes [58,59].

Subjects susceptible to gaining excess fat mass may carry genetic variants that affect the hunger center, adipogenesis, lipid metabolism, insulin signaling and inflammation. The obesity phenotype has been directly and indirectly correlated to more than 600 SNPs [60].

A further example is the polymorphism of glutathione peroxidase, a selenium-dependent enzyme, which has an antioxidant action [61]. The codon 198 polymorphism of human glutathione peroxidase results in a substitution of a proline (Pro) with a leucine (Leu). Researchers have shown that people with the Pro/Leu genotype have an 80% higher risk of suffering from lung cancer, while the Leu/Leu genotypes have 130% more risk than those of individuals with Pro/Pro genotype [62].

The risk of developing breast cancer is also correlated to a specific SNP. In this case, the polymorphism substitutes an alanine in the place of a valine, within the mitochondrial enzyme manganese superoxide dismutase (MnSOD), which is responsible for detoxifying reactive oxygen species (ROS) [63].

Nutrigenomics is concerned with the effects of nutrients and their impact on gene expression levels, proteins and metabolites. In fact, nutrients and food components can influence and regulate the activity of genes both directly and indirectly, even as ligands of transcription factors.

From the biological point of view, nutrigenomics plays a key role in systems biology [64]. On the molecular level, the evaluation of the effects of nutrigenomics requires an accurate evaluation of three interconnected systems: nutrient–nutrients, nutrient–genes, gene–gene interactions [63].

Nutrigenomics, through the study of the genome, allows us to understand the interaction between nutrients and the metabolic pathways for homeostatic control.

It has been shown that some nutrients are capable of modulating the processes of inflammation, angiogenesis and proliferation at the basis of cancer [65,66].

Nutrigenomics studies have led to the important discovery that the transcription of the 3-hydroxy-3-methylglutaryl-CoA reductase (HMGCR) gene can be inhibited by dietary cholesterol and that the transcription of the platelet-derived growth factor and IL1β genes can be reduced by ω3 long-chain polyunsaturated fatty acids (PUFAs) [63]. Epidemiological studies have shown that PUFAs, in particular ω6 fatty acids, can modulate the gene expression of various enzymes involved in the metabolism of lipids and carbohydrates.

In subjects with the less common V162 allele, a marked reduction in the concentration of triacylglycerols can be obtained by elevated intake of ω6 PUFAs. However, this association is not observed in subjects with the L162 allele [67].

The cardioprotective role of ω3 fatty acids is attributed to different mechanism such as lipid metabolism and blood pressure normalization, stabilization of atherosclerotic plaques and anti-inflammatory and anti-arrhythmic effects.

Lipid mediators that can derive from ω6 PUFAs, include the pro-inflammatory prostaglandins and leukotrienes and the anti-inflammatory lipoxins (LX) such as LXA4.

ω3 PUFAs, on the other hand, generate different types of molecules (resolvins, protectins and maresins). Given the powerful actions of these molecules in human disease models, deficiency in the resolution pathways can contribute to many diseases, including atherosclerosis. For this reason, these mediators may become new therapeutic targets for the treatment of inflammatory diseases [68].

A key role in inflammation is played by the NFkB/Rel family, which is capable of inducing nuclear translocation of cytoplasmic complexes and transcription of pro-inflammatory genes [69].

PUFAs and their metabolites can interact directly with a number of key transcription factors, such as NFkB. Through these pathways, it has been seen that the ω3 and ω6 PUFAs influence the gene expression related to inflammation, proliferation and angiogenesis in different ways and can therefore modulate tumorigenesis [70].

Another example of how genetic expression can be modified by nutrients is evident when are exanimated the metabolic pathways of folic acid (vitamin B_9_), cobalamin (vitamin B_12_) and homocysteine [71]. Several studies in mammals demonstrate that, vitamin B_12_ and folate converge at a single metabolic junction involving homocysteine detoxification. Vitamin B_12_ acts as cofactor in a key reaction involving methionine synthase (MS) and folate [72]. The result of this reaction is the formation of methionine from homocysteine. Vitamin B_12_ acts as the catalyzer of the above-mentioned reaction, increases mRNA production, improves translation and heightens post-translational protein stability. This effect does not imply an alteration in protein or mRNA turnover rates, but shifts mRNA from the ribonucleoprotein to the polysome pool, inducing an up-regulation of MS activity. Thus, it was showed that vitamin B_12_ supplementation enhances flux of homocysteine through the MS-dependent transmethylation pathway [71]. A folate and vitamin B_12_ rich diet accelerate homocysteine catabolism, reducing its plasmatic levels [73]. Therefore, the risk of developing hyperhomocysteine-related pathologies results diminished.

The *Dnmts* are a family of enzymes (including *Dntm1, Dntm3a, Dntm3b*) that catalyze the transfer of methyl groups from S-adenosyl-methionine (SAM) to cytosine residues in DNA, thus producing 5-methylcitosine and S-adenosylhomocysteine (SAH). Nutrient supply appears to play a key role in regulating *Dnmt* activity. Selenium (Se) is a microelement present in food and it is known to have potential anti-cancer properties. Interestingly, increased selenium concentrations were found to inhibit *Dntm1* activity and to decrease *Dntm1* protein expression in vitro [74], highlighting the role of dietary factors in methyl utilization and DNA methylation modifications. Some authors report that rising the amount of Se in the diet can increase the expression of the genes involved in synthesizing glutathione peroxidise [75].

Zinc is required for the expression of certain proteins that are involved in regulating gene transcription (i.e., transcription factors), as well as metal transcription factor 1. The amount of zinc in cells is a very sensitive indicator of the expression of a particular gene [76,77,78]. Natural bioactive compounds present in fruits and vegetables, such as flavonoids, phenols, isothiocyanates, indoles and selenium, can prevent carcinogenesis by modulating detoxifying enzymes [79]. Flavonoids, isothiocyanates, vitamin C and E, in fact, are able to trigger the repair of oxidative DNA damage. Isothiocyanates can also regulate the expression of p21, inhibiting the passage from the G2 to the M phase of the cell cycle [80].

## 6. Epigenetics and Epigenomics

Epigenetics is the discipline that studies the mechanisms of gene activation/inactivation, meaning the causal relationship between genes and their products, i.e., protein [81]. Epigenetic events concern DNA modifications which occur before transcription (i.e., mRNA development).

Epigenetic processes make reversible changes in chromatin structure and modify DNA without changing the nucleotide sequence. These processes include DNA methylation and acetylation, as far as histone methylation, phosphorylation and ubiquitination [82]. Bioactive food components may modulate gene expression via epigenetic mechanisms. Histone acetylation is an important epigenetic modification that alters chromatin structure by disrupting its compaction and high-order folding, thus promoting the solubility of chromatin at physiological ionic strength [83]. Acetylation of 46% of maximal site occupancy is enough to inhibit RNA polymerase III transcription. Higher-order chromatin organization can be destabilized by the acetylation of core histone tails, therefore, facilitating transcription. Studies revealed that among the fatty acids, acid butyrate is the most effective inhibitor of histone deacetylase (HDAC), which stimulates or represses the expression of certain genes, causing the stop of cellular proliferation. HDAC activity is inhibited by butyrate, via unknown mechanisms and binding sites. The butyrate responsive elements, located in the promoters of butyrate-responsive genes, mediate the action of butyrate through the Sp1 and Sp3 binding sites. As a consequence of the inhibition of Sp1/Sp3-associated HDAC activity, histone hyper-acetylation occurs. There is also transcriptional activation of the p21waf1/cip1 gene, which arrests cell cycling and proliferation via the action of cyclin dependent kinase 2 activity, in a p53 independent process [84].

HDAC inhibitors act through a zinc binding site. Recent studies have identified a new class of HDAC inhibitors, having short chain fatty acid tethered to a zinc chelating moiety through hydrophobic linkages [85].

Other epigenetic processes involve transcription inhibitors or promoters, such as specific DNA sequences, regulative elements (i.e., enhancers) and polypeptides containing peculiar motifs that directly interact with DNA. The most common motifs are the following: “zinc finger”, “helix-loop-helix”, “leucine zipper” and “high mobility group box” [86]. Other post-transcriptional pathways exist besides methylation, such as protein hyperphosphorylation (another key for anti-proliferative phenomena) [87].

Epigenetic modifications can derive from DNA methylation carried out by the enzyme methyltransferase, which adds methyl groups to the cytosine in the C5 position of CpG dinucleotides. Methylation often causes gene silencing by preventing the binding of transcription factors through the universal methyl donor SAM [88].

Epigenetic processes of DNA methylation-demethylation play an important role in the inhibition or activation of genes. More specifically, methylation is an event that permits the repression of methylated DNA transcription resulting in the maintenance of genes in the inactive state. From DNA analyses, it was found that one in every 100 nucleotides contain an added methyl group, linked with fifth carbon of a cytosine. At this epigenetic level, genes are susceptible to changes that may occur even through the interaction with environmental factors, such as diet. It was showed that dietary factors may influence methylation processes in different ways [89]. For instance, folate, vitamin B12 and vitamin B6 may limit the availability of methyl groups for DNA methylation. Furthermore, bioactive food components (BFC) may influence reactions catalyzed by the methyltransferase enzyme and modify DNA demethylation activity. DNA methylation patterns may even be able to influence responses to BFC by altering their capability of interacting with molecular targets [89].

## 7. Proteomics and Metabolomics

Proteomics determines the protein composition in a cell, organ or organism at a given time. Unlike the genome, the proteome is more dynamic due to post-transcriptional changes. Furthermore, alternative splicing and post-translational modifications make the proteome even more complex.

Over 40,000 genes in the human genome have been recognized to be capable of transcribing and permitting the synthesis of more than 500,000 proteins [7]. Proteomics focuses on the global analysis of these proteins, which may provide a fingerprint of the human body.

In this context, the definition of disease assumes a different connotation, identified as a lack in protein stability [7].

It was found that many proteins have different effects downstream and it depends on of the ligand to which they are linked from time to time. Proteins can carry out different cell activities (i.e., pleiotropism). These activities can differ based on the position of the cell with respect to other cells (i.e., autocrine, paracrine, endocrine effects), or with respect to their activated-inactivated state. For these reasons, these types of proteins were defined “moonlighting proteins” [90,91].

Metabolomics is the discipline that also considers other low molecular weight products (besides proteins) and their roles as metabolites. It deals with quantification of metabolome dynamic properties and incorporates information about measurement of genetic and proteomic responses to nutritional factors [7].

In contrast with the genome, but similarly to the proteome, the metabolome is dynamic over the time [7]. Genetic expression is not comparable to metabolism [14]. The metabolome’s extreme variability according to age, hormonal status and/or health, may find a solution in completely and quantitatively phenotyping nutrients and metabolic intermediates. Young proposed to shift the target to stem cells, which may allow the definition of individual nutrient requirements. This approach may maximize specificity of nutrient action in terms of improved resistance to diseases, longevity and physical performances [7,92].

Noguchi et al. [93] showed metabolomes mechanism action of and the possibility of measuring their concentration changes in an organism through amino acids study. After proteins and amino acids ingestion, there is a metabolic adaptation. There are changes in genetic expression of enzymes’ activity, which affect metabolism of districts/organs/systems. These variations may be measured in order to furnish precise information about intake. In contrast, changes of plasma amino acids concentration over time after ingestion of a meal, doesn’t reflect real dietary amino acids intake [94,95,96]. Amino acids control levels are several and their expression and functions of great heterogeneity can be grouped in cluster analysis of multivariate correlation (CAMC) [93]. CAMC allow for group quantitative analysis on known metabolites data and make it easier to extrapolate information concerning the strict relation between metabolites behavior and toxicological parameters associated with amino acids intake increase [93].

Finally, capability in predicting consequences of food intake in welfare and well-being balance depends on the knowledge of metabolites kinetics, enzymatic reactions and pathways.

## 8. From Disease to Health Status

Diet recommendations should take place in a safe environment and prophylaxis plans should aim to exploit potential health promoting factors in foods, reaching socioeconomic benefits.

The balance between health and disease can be influenced by dietary chemicals components. As a matter of fact, diet and lifestyle represent important risk factors for several CDDs [97].

Several BFC have antihypertensive, antioxidant, antithrombotic, hypocholesterolemic, hypotriglyceridemic and anti-obesity effects [61,98]. It has been demonstrated that a correct lifestyle, particularly based on the correct intake of nutrients and bioactive foods, is correlated to a low risk of morbidity/mortality and a much longer life expectancy [99,100,101]. In particular, MD is associated with a delay in aging/death through the supply of antioxidant molecules and the reduction of inflammatory cytokines associated with several diseases, e.g., CVDs [61,102,103,104].

A diet rich in fruits and vegetables has shown to have cardio-protective effects in several epidemiological studies. CVDs represent an important cause of mortality and morbidity worldwide. Moreover, some prospective studies show an inverse correlation between fruit and vegetable assumption and the development of CVDs (like myocardial infarction and stroke).

The reduction in CVD risk may be due to the fiber, potassium and folate contained in many nutrients and phytochemicals in fruits and vegetables [105,106].

Many studies recommend a healthy lifestyle based on physical exercise and a diet rich in fiber, antioxidants, ω-3 PUFAs, nutraceuticals, vitamins and minerals, in order to prevent CVD [107,108].

It has been demonstrated that a daily intake of ω6 and ω3-fatty acids decreases the biosynthesis of fatty acids, the triglyceride production and secretion in the liver. Furthermore, they reduce the levels of fatty acid oxidation within the liver and skeletal muscles [109,110].

In order to influence gene expression and metabolism, the ω6 and ω3 fatty acids have to be metabolized in the delta-6 desaturase pathway, into highly PUFA.

The risks associated with high triglyceride levels (e.g., accelerated apoptosis), may be decreased following a MD rich in olive oil and marine and vegetable lipids (e.g., four parts olive oil, one part fish oil and one part vegetable oil). This diet may even delay the onset of T2DM [111,112,113].

n-3 highly unsaturated fatty acids (HUFAs) can regulate lipid metabolism, improve insulin sensitivity and increase nonoxidative glucose metabolism, but these beneficial effects depend directly on the amount of PUFA consumed in the diet [114,115].

The consumption of fruits and vegetables is high in the MD and several of its main components, such as olive oil, red wine and specific plants (Thymus piperella, Berberis vulgaris, Scandix australis, Vitis vinifera, Foeniculm volgaris, Borago officinalis, Lactuga viminea, etc.) have attracted the interest of the scientific community. These elements have been studied from a pharmacological perspective [116]. For this reason, epidemiological studies have investigated the impact of various elements of MD on longevity and health, preventing chronic and age-related diseases [104,117].

In this contest, unsaturated fatty acids, polyphenols and various vitamins (such as vitamin E and C), have shown to produce positive effects on human health [118,119].

Pharmacologically, polyphenols can act directly or indirectly. Their antioxidant effects include direct scavenging of free radicals and ROS, while their indirect antioxidant activity involves enzyme inhibition. Polyphenols may also show anti-inflammatory, anti-proliferative, anti-diabetic and expression-modifying actions [120].

MD is able to reduce the levels of lipids in the blood stream and it also has a protective role against oxidative stress [116]. Oxidative damage is thought to represent one of the main factors leading to CDDs such as atherosclerosis and cancer. Several studies suggested the direct correlation between dietary fruit and vegetable consumption and the amounts of antioxidant vitamins (ascorbic acid, tocopherol and carotenoids) in plasma and the risk of death from cancer or coronary heart diseases [121]. The MD is characterized by a high content of antioxidant compounds and it is also able to modulate the levels of oxidative stress [122,123]. The oxidized or oxidizing compounds can be counteracted by an high intake of antioxidant substances as well as by the fiber [124].

The study of the modulation of the inflammatory state through nutrigenomics would seem to identify the MD as preventive for CDDs [125,126]. Some components of the MD, such as ω3, are known to alter the expression of chemical mediators of the inflammatory state [117,127].

The course of some CDDs seems to be influenced by genes encoding cytokines such as IL-1, IL-6 and TNFα, which in turn are able to modulate the gene expression of inflammatory mediators [128]. The nutritional products could be used as efficient preventive agents on the generic variations that cause an altered inflammatory state. Once the mechanism behind these interactions is understood, nutrients could be applied to large segments of the population [129]. There is a plethora of bioactive substances in plant used in the MD, as flavonoids and polymeric flavonoid, carotenoids, monophenolic alcohols, monoterpens, phenolic acids, tannins and others with anti-inflammatory properties. The ability of plants extracts to inhibit inflammatory cytokine stimulation, such as TNF-α, IL-8, IL-1 and IL-6, chemokines, cell adhesion molecules and iNOS-dependent synthesis of nitric oxide, explains the anti-inflammatory effects of MD [130,131]. Complex carbohydrates, specifically fiber, can influence the inflammatory state. A recent study has shown that the fiber content in the meal can affect circulating IL-18 levels in both healthy subjects and patients with T2DM [132].

Given the anti-inflammatory effect given by dietary fiber, it is possible to hypothesize that transient oxidative stress may be influenced by the fiber present in the diet. In addition to the anti-inflammatory role given by the dietary fiber, ω3 fatty acids have also been suggested to play a positive role in the inflammatory state, as well [107,133]. Moreover, ω3 fatty acids can reduce the risk of coronary heart disease (CHD) by lowering serum triglyceride levels and ameliorating endothelial disfunction [134]. α-linoleic acid (ALA), an ω3 fatty acid high in canola, flaxseed, soybean oils, can be converted to eicosapentaenoic acid (EPA) and docosahexaenoic acid (DHA), thus preventing CHD by decreasing thrombotic tendency [135,136].

Since metabolic syndrome is characterized by a low-grade inflammatory state with a simultaneous increase in fat mass as well as in the syndrome of normal weight obese (NWO) and obesity, the MD based on a large consumption of whole grain cereals, fresh fruit, vegetables, legumes, dried fruit (such as walnuts) and extra virgin olive oil is a useful tool in reducing the prevention of overweight, obesity and cardiovascular risk, at the same time reducing the incidence of CDDs [137].

Both the DASH-diet (Dietary Approaches to Stop Hypertension) and the OmniHeart diet (Optimal macro-nutrient intake to prevent Heart disease) based on reduced intake of saturated fats and an increase supply of fresh fruits and vegetables, with other specific characteristics of each, showed an improvement on blood pressure and lipid level obtaining a reduced CVD risk [138].

Angiotensin converting enzyme (ACE) inhibitory peptides from fish, soy, egg, meat and vitamins, polyphenols, carotenoids from fruits and vegetables, antioxidant peptides from milk casein, whey protein and soy protein, antithrombotic peptides from bovine K-caseine, hypocolesterolemic proteins from whey, can reduce the risk of CVD [98].

Dietary modifications may be particularly useful in subjects predisposed for neurodegenerative diseases with high homocysteine levels and δ4 allele of apolipoprotein E, through providing antioxidants, anti-inflammatory agents, supplying vitamin B6, B12, folic acid and reducing saturated fats and cholesterol [139,140]. Both Alzheimer and Parkinson diseases are characterized by oxidative stress, metabolic impairment and abnormal protein aggregation, which means a need for antioxidants and agents that preserve mitochondrial function and a detoxifying system more efficient than in normal subjects without a genetic predisposition [141]. However, although no evidence shows any clear benefit of neurodegenerative disorders by the use folic acid and by reducing food intake, it will be of considerable interest to perform this kind of trial before the onset of disease or at early preclinical stages, as a prevention intervention [141].

BFCs not only act as buffering molecules on oxidative stress and metabolic impairment, but they act also as key-elements in human physiology [142].

Oltvaiand Z.N. and Barabasi A.L. represented the complexity of cellular processes as a pyramid; the base is the cellular fundamental molecular components and the apex constituted of phenotypic characteristics [143]. It was demonstrated that cells with molecular components as genes, mRNA, proteins and metabolites are in close relationship with environmental factors. There is a one-to-one relationship between cellular events and BFC, which represents the true nutritional homeostasis [89,144].

## 9. Gut Microbiota and Personalized Nutrition

The gut microbiota is the “set” of microorganisms colonizing the intestine in humans [145]. It has been estimated to be around 10^14^ resident microorganisms and its activity is influenced by variables such as age, gender, CDDs and ethnicity [146,147,148].

The balance of its composition is important for maintaining the state of health. In fact, a large number of studies showed that the alteration of the Firmicutes/Bacteroides ratio can cause the development of metabolic pathologies such as central obesity and T2DM [125]. Also host gene expression patterns, eating habits, physical activity, drugs and others lifestyle factors can alter the gut microbiota abundance and its α-diversity [148,149,150,151,152,153].

All macronutrients (proteins, fats and carbohydrates) perform an action on the gut microbiota causing a change in its composition and this change can have positive or negative effects on host health. For example, the consumption of animal protein decreases short chain fatty acids (SCFAs) which in turn cause inflammatory bowel disease (IBD) and increase the CVD risk. Conversely, the consumption of plant protein is associated to a high microbial α-diversity through an increased production of SCFA which have anti-inflammatory activity and contribute to gut mucosa integrity [154,155].

Consumption of saturated fat increases insulin resistance and white adipose tissue (WAT) inflammation [156]. On the contrary, unsaturated fat decreases the inflammation of WAT, total cholesterol (TC) and low-density lipoprotein (LDL) cholesterol [17,123,145,157].

Recent studies highlighted that consumption of digestible carbohydrates of natural origin have a less negative effect on health, compared to artificial ones. More specifically, artificial digestible carbohydrates cause an enhancement in intestinal dysbiosis and carbohydrate intolerance [145,158]. Instead, non-digestible carbohydrates such as resistant starch and vegetable fiber are not enzymatically split in the small intestine and for this reason, they can reach the large intestine, where they permit the fermentation of the gut microbiota [145]. In this context, non-digestible carbohydrates act as prebiotics stimulating the growth of some species that contribute to host health [159]. Several researches showed that prebiotics are able to influence metabolic and immune markers such as IL-6, insulin resistance and post-prandial blood glucose peak [160,161,162]. These data highlight that gut microbiota is able to modulate the metabolic and immune response of the host according to the diet, thus contributing to the maintenance of host health status.

In the last decade, the modulation of intestinal microbiota through probiotic, prebiotic, antibiotic and postbiotic intake has aroused considerable interest in the scientific community [163]. This therapeutic approach would seem to be highly effective for personalized nutritional interventions. In fact dietary patterns or specific foods can alter the presence of different types of bacteria in the gut, which in turn can affect health [164]. Several in vivo studies, both on animal models and in humans, have attempted to explain the influence of the microbiota on health status. Animal models can help to understand the mechanism that regulates microbiota composition and its interactions with the environment and then transfer this knowledge to humans [165].

In support of this revolutionary discovery, a study conducted by Zeevi et al. [166] has developed a machine-learning algorithm able to predict levels of postprandial blood glucose through the integration of clinical data such as blood parameters and composition of the intestinal microbiota. The study involved 800 T2DM subjects and lasted 7 days. This interesting application has been shown to be useful in decreasing postprandial glucose levels and the usefulness of such personalized intervention should be studied for a longer period and on a larger population.

In light of these new evidences, the crosstalk between diet, gut microbiota and health paves the way for new therapeutic strategies that act on health, through the modulation of eating habits. The modes of interaction between microbiota and host are different, among these we find: the modulation of the host’s immune system [167], metabolic health [168] and the promotion of certain bacterial species growth [169]. The possibility of intervening in pathological conditions through the variation of the diet, aimed at improving the state of health is of considerable interest and increasingly feasible [170]. These interventions could be addressed to manage CDDs such as T2DM and obesity. The field of precision nutrition could represent a new approach for the management of diseases with a strong genetic component. Thanks to new discoveries, precision nutrition should address not only the host but also to its gut microbiota, that is able to guarantee its health.

Recent data show that personalized nutrition plays a pivotal role in preventing the development of CDDs and interactions of food with the genome and the microbiome could become the new challenge for the future in preventive medicine [171]. It will therefore be necessary to develop new tools that can customize nutrition based on the microbiome and individual genetics.

## 10. Conclusions

With the advancement of -omics techniques, the definition of food has changed since it no longer represents only a source of energy and macronutrient and microelements, but an important factor able to determine the quality of health. Foods, besides complying with their primary function of energy source, will be more and more selected on the basis of bioactive food components. BFC will be similar to drugs in disease treatment. Foods and drugs won’t only be remedies of an unsteady condition, but they will be a part of global background of prophylaxis or even treatment of diseases, particularly for CDDs.

Many pathophysiological phenomena can be explained by the relationship between micronutrients and gene expression that can also represent a primary aim in delaying the onset of CCDs.

From gene study, through genomics, genetics and epigenetics, with the fundamental support of nanotechnologies and bioinformatics and related with proteomics and metabolomics, it will be possible to obtain complete notions on cell-environment and definitely organism-nutrient interrelations.

A better knowledge on nutrient–gene–metabolite pathways will permit to act in symbiosis at various levels of cellular study. In this context, the possible interactions between gut microbiota and food should be evaluated.

Personalized nutrition should select the most suitable foods for each individual, for example based on the action they have on gene expression and/or the composition of gut microbiota.

By only taking into consideration personal needs and characteristics, besides the individual’s genetic profile, it is possible to develop dietary protocols that are more likely to be useful and successful.

## Figures and Tables

**Table 1:** Gene polymorphism and nutrients to supply for personalized diet.

Gene	Activity	Disease	Nutrients to Supply	Reference
FTO	Hunger keeper Energy homeostasis Fat mass storage	Obesity	MD	[24]
IL-6	Inflammation	CCD CVD Obesity	MD Polyphenols Dried Fruits	[28]
MCR4	Leptin pathways	Obesity	MD	[23]
MTHFR	Homocysteine metabolism Lean mass development	CVD CDD Obesity Sarcopenia	Folate Cobalamin Protein	[33,34]
TNFα	Inflammation	CCD CVD Obesity	MD Polyphenols Dried Fruits	[36,37,38]
GSTM1	Oxidative Stress	Non-Small Cell Lung Cancer	Antioxidant compound	[62]
MnSOD	Oxidative Stress	Breast Cancer	Antioxidant compound	[63]
HMGCR	Metabolism of lipids and carbohydrates	CDD	MD	[63]
IL-1β	Metabolism of lipids and carbohydrates	CDD	ω3 long-chain polyunsaturated fatty acids (PUFAs)	[62]
NFkB	Inflammation, Proliferation Angiogenesis	CDD Cancer	ω3 ω6 PUFAs	[70]

MD: Mediterranean diet, CVD: Cardiovascular disease; CDD, Chronic-degenerative disease, PUFA: Polyunsaturated fatty acids; MTHFR: Methyltetrahydrofolate reductase; FTO: Fat mass and obesity-associated; MnSOD: Manganese superoxide dismutase; HMGCR: 3-Hydroxy-3-methylglutaryl-coa reductase gene; MCR4: Melanocortin-4 receptor; TNFα: Tumor necrosis factor-α; NFkB: Nuclear factor kappa-light-chain-enhancer of activated B cells; IL: Interleukin; GSTM: Glutathione S-transferase mu.

**Table 2:** Nutrients activity and effects.

Nutrients	Activity	Effect	Reference
Folic acid (B9) Cobalamin (B12) Pyridoxine (B6)	Cofactor methionine synthase Availability of methyl groups for DNA methylation	Homocysteine detoxification Epigenetics regulation	[72,73,139,140]
Selenium	Inhibit of Dntms	Anti-Cancer	[74]
Ω-3 and 6 α-linoleic acid	Lowering serum triglyceride levels Ameliorating of endothelial disfunction Anti-inflammatory	Blood pressure normalization Stabilization of atherosclerotic plaques	[68,134,135,136]
Zinc	Metal Transcription Factor 1	Anti-Cancer	[76,77,78]
Isothiocyanates	Expression of p21 Inhibit of passage from the G2 to the M phase of the cell cycle	Anti-Cancer	[80]
Acid butyrate	Inhibitor of histone deacetylase Inhibition of Sp1/Sp3 Activation of the p21waf1/cip1	Anti-Cancer	[84]
MD (Fiber, Vitamins, Antioxidant compound, Polyphenols, ω3-6, Olive oil, Fish oil, PUFAs, Minerals)	Decreases the biosynthesis of fatty acids and the triglyceride production Levels of fatty acid oxidation	Prevent of CVD and CDDs Prevent damage of skeletal muscles	[104,105,106,107,108,109,110,111,112,113,114,115,116,117]
	Antioxidant effects Direct scavenging of ROS	Prevent of CVD and CDDs Anti Cancer	[120,125,126]
Plant used in the MD (flavonoids and polymeric flavonoid, carotenoids, monophenolic alcohols, monoterpens, phenolic acids, tannins)	Inhibit inflammatory cytokine stimulation, cell adhesion molecules and iNOS-dependent	Prevent of CVD, CDDs Anti Cancer	[130,131]
Plant protein	production of SCFA	High microbial diversity Gut mucosa integrity	[155]

MD: Mediterranean diet, CVD: Cardiovascular disease; CDD, Chronic-degenerative disease, NOS: Nitric oxide synthase; PUFA: Polyunsaturated fatty acids; p21waf1/cip1: Cyclin-dependent kinase inhibitor; ROS: Reactive oxygen species; SCFA: Short chain fatty acids; Sp: Specificity protein.

## Abbreviation

4P	Predictive: Preventive, Personalized and Proactive
ACE	Angiotensin-Converting-Enzyme
ALA	α-Linoleic Acid
BFC	Bioactive Food Components
BMI	Body Mass Index
CAMC	Cluster Analysis of Multivariate Correlation
CDD	Chronic-Degenerative Disease
CHD	Coronary Heart Disease
CSF	Cerebrospinal Fluid
CVD	Cardiovascular Diseases
DASH	Dietary Approaches to Stop Hypertension
DHA	Docoashexaenoic Acid
Dnmt	DNA Methyltransferase
EPA	Eicosapentaenoic Acid
FTO	Fat Mass and Obesity-Associated
GPS	Genome-Wide Polygenic Score
GSTM	Glutathione S-transferase Mu
GWAS	Genome-Wide Association Study
HDAC	Histone Deacetylase
HMGCR	3-Hydroxy-3-Methylglutaryl-Coa Reductase Gene
HUFA	n-3 highly unsaturated fatty acids
IBD	Inflammatory Bowel Disease
IL	Interleukin
iNOS	Inducible Nitric Oxide Synthase
LAGB	Laparoscopic Adjustable Gastric Banding
LDL	Low-Density Lipoprotein
LX	Lipoxins
MC4R	Melanocortin- 4 Receptor
MD	Mediterranean Diet
MnSOD	Manganese Superoxide Dismutase
MS	Methionine Synthase
MTHFR	Methyltetrahydrofolate Reductase
MTRR	Methionine Synthase Reductase
NFkB	Nuclear Factor Kappa-Light-Chain-Enhancer of Activated B Cells
NWO	Normal Weight Obese
OmniHeart	Optimal Macro-Nutrient Intake to Prevent Heart Disease
PUFA	Polyunsaturated Fatty Acids
ROS	Reactive Oxygen Species
SAH	S-Adenosylhomocysteine
SAM	S-Adenosyl-Methionine
SCFA	Short Chain Fatty Acids
Se	Selenium
SNPs	Single Nucleotide Polymorphisms
T2DM	Type 2 Diabetes Mellitus
TC	Total Cholesterol
TNFR2	Tumor Necrosis Factor Receptor-2
TNFα	Tumor Necrosis Factor-α
WAT	White Adipose Tissue
